# High resolution paddy rice maps in cloud-prone Bangladesh and Northeast India using Sentinel-1 data

**DOI:** 10.1038/s41597-019-0036-3

**Published:** 2019-04-11

**Authors:** Mrinal Singha, Jinwei Dong, Geli Zhang, Xiangming Xiao

**Affiliations:** 1 Key Laboratory of Land Surface Pattern and Simulation, Institute of Geographic Sciences and Natural Resources Research, Chinese Academy of Sciences, Beijing, 100101 China; 2 College of Land Science and Technology, China Agricultural University, Beijing, 100193 China; 3 Department of Microbiology and Plant Biology, Center for Spatial Analysis, University of Oklahoma, Norman, Oklahoma 73019 USA

**Keywords:** Climate change, Environmental impact, Agriculture

## Abstract

Knowledge of where, when, and how much paddy rice is planted is crucial information for understating of regional food security, freshwater use, climate change, and transmission of avian influenza virus. We developed seasonal paddy rice maps at high resolution (10 m) for Bangladesh and Northeast India, typical cloud-prone regions in South Asia, using cloud-free Synthetic Aperture Radar (SAR) images from Sentinel-1 satellite, the Random Forest classifier, and the Google Earth Engine (GEE) cloud computing platform. The maps were provided for all the three distinct rice growing seasons of the region: Boro, Aus and Aman. The paddy rice maps were evaluated against the independent validation samples, and compared with the existing products from the International Rice Research Institute (IRRI) and the analysis of Moderate Resolution Imaging Spectroradiometer (MODIS) data. The generated paddy rice maps were spatially consistent with the compared maps and had a satisfactory accuracy over 90%. This study showed the potential of Sentinel-1 data and GEE on large scale paddy rice mapping in cloud-prone regions like tropical Asia.

## Abstract (summary)

**Table:** 

Design Type(s)	image analysis objective • observational design
Measurement Type(s)	rice field
Technology Type(s)	satellite imaging
Factor Type(s)	season • geographic location
Sample Characteristic(s)	Bangladesh • paddy field • India

Machine-accessible metadata file describing the reported data (ISA-Tab format)

## Background & Summary

Paddy rice agriculture occupies more than 12% of global cropland area^1^. As a major crop, rice contributes a significant portion to the global food supply. Rice stands as a staple grain for more than 50% of the world’s 7.5 billion people^2^. Rice agriculture plays a key role not only to the global food security but also to the environmental and health issues such as the freshwater uses^3^, global warming^4,5^, and highly pathogenic avian influenza virus transmission^6,7^. In recent years, paddy rice cultivated areas have become increasingly threatened by the changing patterns of precipitation, global temperature rise, rapid urbanization and industrialization. This has resulted in utilizing the limited lands with the intensifying cropping cycle^8^ and increased uses of water resource to enhance the production. The monitoring of these increasing rice agricultural intensification will be of significance for decision making supports related to food, water and health security as well as the mitigation and adaptation of climate change.

India and Bangladesh are respectively the second and fourth largest rice producer country in the world^1^, characterized by multiple rice cropping systems. However, time to time these nations import rice to meet the demands and food security is still a major concern for the two countries. Thus, accurate mapping and monitoring of paddy rice is essential in the region to ensure food security, sustainable development, and socio-economic development. Recognizing the role of rice agriculture in food security and the environment, several efforts have been initiated to monitor rice agriculture such as the Asian Rice Crop Estimation and Monitoring (Asia-RiCE)^9^ and the Group on Earth Observations Global Agriculture Monitoring (GEOGLAM)^10^.

International Rice Research Institute (IRRI) freely distributed paddy rice maps^11^ for Asia (for the year of 2000–2001), Nepal (2000–2009), Bangladesh (2010), Philippines (2000–2012), South Asia (2000–2001) and for the Indian state of Tamil Nadu (2001–2011) and Odihsa (2000–2010). These maps are of coarse spatial resolution with 500 m and more than seven years old. Recent and high spatial resolution paddy rice maps are rarely available for the scientific communities particularly for cloud-prone tropical regions in Bangladesh and Northeast India with newly increasing planting areas and cropping intensity^12^.

Several techniques and approaches were used to map paddy rice using optical remote sensing data^13^. However, reliability of the applied algorithms depends on the availability of images during the key paddy rice growth stages such as the rice planting and flooding phase. The rice transplanting phase coincides with the rainy season with frequent cloud cover in the tropical regions where paddy rice mostly grows. Traditionally, large scale paddy rice mapping was conducted using the optical satellite imagery of high temporal resolution at coarse spatial resolution ranging from 500 m to 1 km^14^. Daily MODIS data can provide more observations due to its higher temporal resolution; however, its moderate spatial resolution cannot capture the small-size fields and mixed agricultural landscape in tropical Asia where smallholders dominated the farming system. Large scale paddy rice mapping at medium or high spatial resolution is rare, primarily due to the limited availability of the cloud-free optical imagery. Existing studies showed its low valid observation availability and accuracy due to frequent cloud coverage^14^. Despite the relatively high revisit cycle of Sentinel-2 (5-day) and Landsat (16-day), there are not enough clear sky observations for effective mapping of paddy rice in tropical regions at high resolution and large area. In a cloud-prone region like Bangladesh, it is not uncommon to have any images in one or two months with good quality observations. The all-weather capable, active microwave sensing system that operates independent of sun illumination make Synthetic Aperture Radar (SAR)^14^ particularly suitable for mapping paddy rice in the cloud-prone tropical and sub-tropical regions.

The SAR-based mapping were limited to small study areas due to the intensive amount of data processing. With the advent of high performance cloud computing resources like Google Earth Engine^15^ (GEE), NASA Earth Exchange^16^, Amazon Web Services^17^, very large geospatial data analysis became possible. However, exploitation of these cloud computing techniques in the remote sensing applications is still in its infancy. Inside GEE, we applied machine learning Random Forest (RF) classifier for the mapping because of RF’s efficient ability to produce accurate classification results and handle large and diverse datasets^18^.

Based on the existing challenges of paddy rice identification at high resolution in large and frequent cloud cover regions, and unavailability of the recent seasonal paddy rice maps, our objective was to generate an unprecedented 10 m paddy rice map for three distinct seasons: Boro, (December/January–April), Aus (April/May–June/July), and Aman (July/August–November) for the entire country of Bangladesh and Northeast India, using GEE and time series Sentinel-1 SAR imagery in 2017.

## Methods

**Fig. 1:**
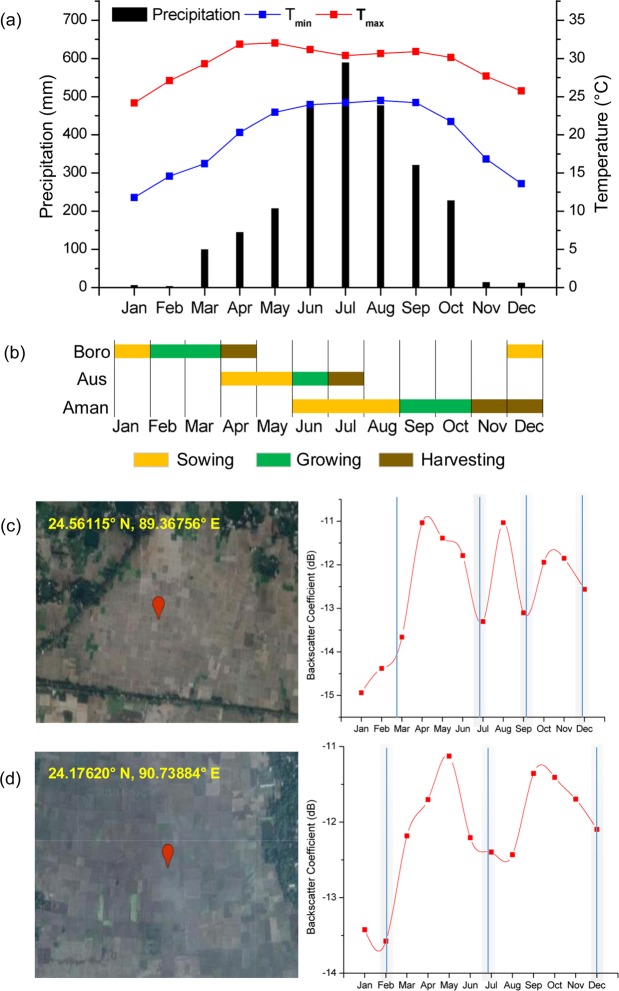
Paddy rice calendar and thrice cropping system in Bangladesh. (**a**) the annual variation of monthly maximum temperature, monthly minimum temperature, and monthly precipitation for 2017 using the Climatic Research Unit (CRU) data; (**b**) crop calendar; (**c**,**d**) temporal profiles of the Sentinel-1 SAR backscatter coefficients for two sites of Bangladesh with triple and double cropping systems.

**Fig. 2:**
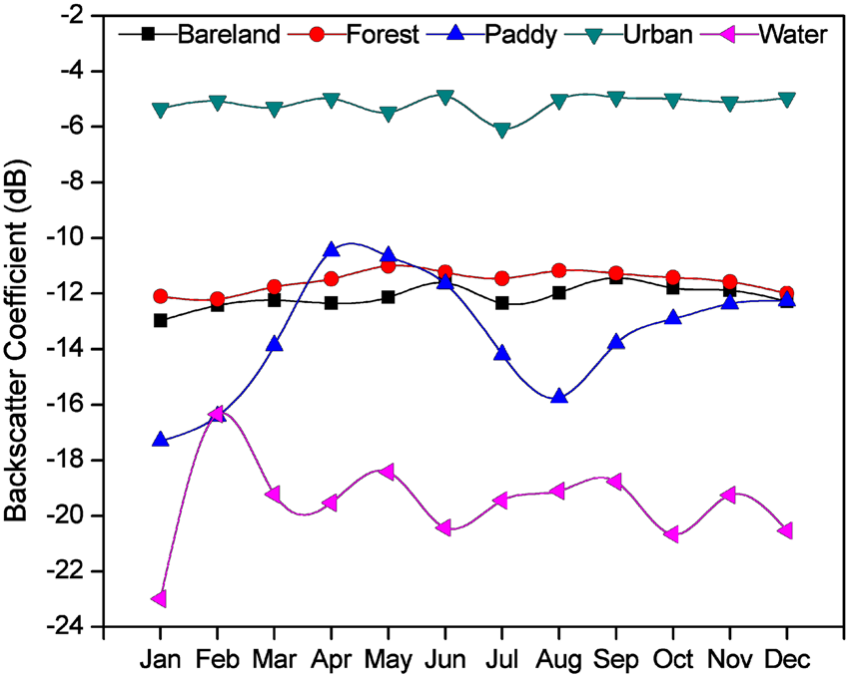
Temporal profiles of backscatter for various land cover classes. The temporal profiles of backscatter values from random locations. The paddy rice class show distinct profile than other classes. The profile was generated from the following random locations; bare land: 23.85662°N, 90.48301°E; forest: 21.94974°N, 89.30164°E; paddy rice: 24.56287°N, 89.34906°E; urban land: 23.79381°N, 90.37183°E; water: 23.73913°N, 90.71758°E.

Sentinel-1 SAR backscatter can be used as an indicator for tracking paddy rice growth as the backscatter values change with the varying conditions of paddy rice stages. Unlike other crops, rice spends a substantial time submerged in water^19^. Paddy rice has three distinct growing stages: the flooding/transplanting period, the vegetative period, and the after-harvest period^20^, these stages provide distinct SAR backscatter values that can be utilised for remote sensing analysis (Fig. 1). Initially, the SAR backscatter values are low for paddy rice during the flooding/transplanting period due to the presence of water, followed by a significant backscatter increase during the vegetative stage due to volume scattering from the rice plant and finally decrease in backscatter during the rice harvest stage due to surface scatter from the open land. For this study, we used the VH (vertical transmit, horizontal receive) polarization data because it is more sensitive to rice growth stages than the VV (vertical transmit, vertical receive) polarization data^21^. The SAR backscatter profiles were generated for random paddy rice sites in the two regions of Bangladesh (Fig. 1). The backscatter profiles were consistent with the rice crop calendar. Also, the number of peaks in the backscatter profiles agreed with the paddy rice intensity. The high yield paddy rice in the Boro and Aman seasons have longer growing periods and the Aus season has the shortest growing period, which are evident according to the backscatter profiles (Fig. 1). The Boro and Aman season paddy rice are cultivated using the water from irrigation and rainfall respectively and the paddy plants stay a long time under water in these seasons, generating a smooth and long backscatter profile. The Aus season paddy rice is cultivated during the short pre-monsoon rainfall period under a relatively dry conditions, which creates a short duration and high peak backscatter, reflecting from the mixture of soil and rice plant. The time series backscatter values of non-paddy rice classes were significantly different from paddy rice class (Fig. 2).

**Fig. 3:**
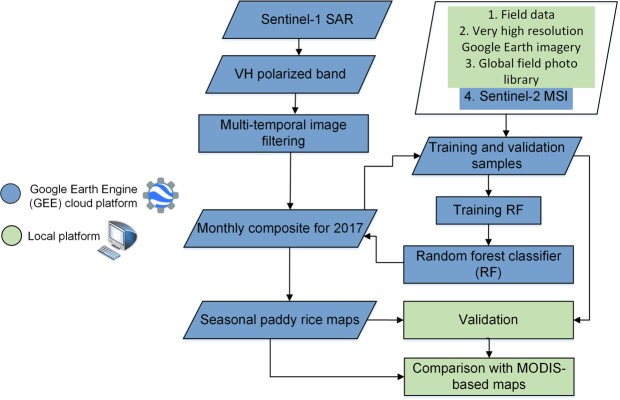
Schematic diagram of workflow in this study. Overview of adopted methodology for paddy rice field extraction. The methods comprise of GEE cloud platform and local processing.

We implemented the random forest^18^ (RF) machine learning algorithm using all the VH polarized SAR data available for 2017 from Sentinel-1 satellite to map paddy rice. The RF algorithm yields high classification accuracy, can successfully handle high data dimensionality, computationally fast, robust to overfitting, and can provide a measure of variable importance^18^. For this study, we selected computationally efficient RF particularly to handle large Sentinel-1 SAR datasets for entire Bangladesh and Northeast India. A comprehensive schematic diagram of the adopted methodology is given in Fig. 3.

### Study area

#### Bangladesh

**Fig. 4:**
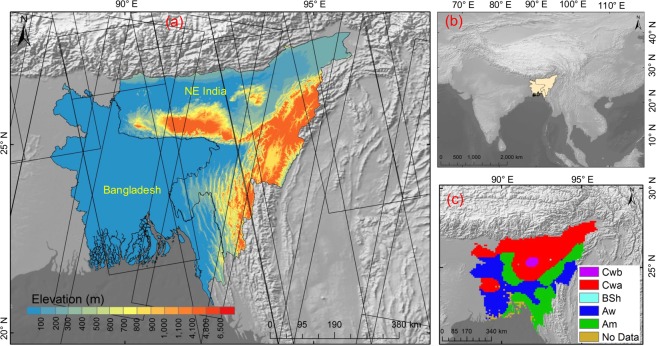
Study area with topographic and climate characteristics. (**a**,**b**) location of Bangladesh and Northeast India; (**c**) climate zone, the climate zone data is from Peel *et al*.^34^, Cwb: subtropical highland, Cwa: humid subtropical, BSh: hot semi-arid, Aw: tropical wet and dry, Am: tropical monsoon. In the left hand image, the black lines are Sentinel-1 orbit path. Background image is shaded relief from elevation data.

Bangladesh is situated in the South Asia (Fig. 4) and the fourth-largest rice producer in the world. It covers a land mass of 144,000 km^2^ and extent from 20°44′00′′ to 26°37′51′′N latitude and 88°0′14′′ to 92°40′08″E longitude. Bangladesh is primarily a flat terrain except the Chittagong Hill Tracts (CHT) regions in the southeast with an average elevation of over 300 m. The country has subtropical monsoon climate with summer, monsoon and winter as the most distinct and prominent seasons^22^. The annual average temperature ranges from 18 °C to 29 °C. The average annual precipitation varies between 200 mm and 2000 mm approximately. About 230 rivers including the three major rivers: Ganges, Brahmaputra and Meghna and their tributaries flow across the Bangladesh down to the Bay of Bengal. Most of the Bangladesh consists of low and fertile land with the flood-plain deltas of these rivers. Agriculture areas cover around 70% of the country’s total land and paddy rice is the primary crop^1^. Paddy rice is cultivated in three distinct seasons in a year in this country mainly in Boro (December/January–April), Aus (April/May–June/July) and Aman (July/August–November/December). Boro is mainly the dry season irrigated paddy rice. Aus is pre-monsoon direct-seeded upland paddy rice. Aman is monsoon season rainfed lowland paddy rice.

#### Northeast (NE) India

NE India is adjacent to Bangladesh (Fig. 4). Except Assam, NE India is primarily hilly and it covered with the eastern Himalaya and Patkai hill ranges. The flat area of Assam consists of Brahmaputra and Barak valley. The elevation of NE India ranges from 50 m to 7000 m. NE India has a subtropical climate. The mean annual precipitation varies from 1500 mm to 2500 mm approximately. The mean annual temperature varies from 5° to 30 °C. Paddy rice is the primary crop of the region accounting about 80% of the total cultivated area^1^. Paddy rice is cultivated one to three seasons in a year in the region. The autumn season rice (Aus) is cultivated during the Mar/April–Jun/July. The summer season rice (Aman) is cultivated in Jun/July–November/December. The winter season rice (Boro) is planted from November/December to April/May. The summer or the monsoon is the primary season for rice due to the abundant rainfall in this time.

### Sentinel-1 SAR data and pre-processing

The Sentinel-1 satellite was launched and operated by European Space Agency and the data are available to public for free^23^. The data are provided in C-band SAR imagery (5.4 GHz) globally with a 12 day or 6 day revisit cycle depending on the availability of Sentinel-1A and 1B imagery^24^. Sentinel-1 collects the imagery in four modes: Stripmap (SM), Interferometric Wide Swath (IW), Extra Wide Swath (EW) and Wave (WV) with varying resolutions, polarizations, extents and purposes. In this study we used the IW mode, which fulfils most current service requirements as this mode avoids conflicts and preserves revisit performance, provide consistent long-term archives and designed to acquire imagery of land surfaces^22^. The SAR imagery of IW mode is provided in dual-polarization with vertical transmit, vertical receive (VV), and vertical transmit, horizontal receive (VH). We used Level-1 Ground Range Detected (GRD) product processed to backscatter coefficient (σ°) in decibels (dB)^25^. The GRD scenes constitute of focused SAR data that has been detected, multi-looked and projected to ground range using the Earth ellipsoid model WGS84^25^. The spatial resolution of this imagery is 10 meter × 10 meter. These SAR data were accessed through the Google Earth Engine (GEE). The Earth Engine pre-processed the Sentinel-1 data to derive the backscatter coefficient in each pixel using the following steps as implemented by Sentinel-1 toolbox^26,27^: (1) apply orbit file, to update orbit metadata with a restituted orbit file; (2) GRD border noise removal, this step removes low intensity noise and invalid data on scene edges; (3) thermal noise removal, this process removes additive noise by reducing discontinuities in sub-swaths for multi-swath acquisition; (4) radiometric calibration, this process calculates backscatter intensity using sensor calibration parameters; (5) terrain corrections using SRTM or ASTER DEM, this process converts the data from ground range geometry to backscatter coefficient (σ°) accounting terrain characteristics; (6) the terrain corrected data were converted to decibels via log scaling (10*log10(x)) and quantized to 16-bits.

Finally, we filtered the backscatter time series using the multitemporal speckle filter^28^, to minimize the influence of environmental conditions and to remove noises existing in the Sentinel-1 datasets due to speckle.

### Random forest classifier

Random Forest (RF) is an ensemble machine learning algorithm proposed by Breiman^29^ for classification. This algorithm is popular in remote sensing community due to its classification accuracy and has been successfully used for many applications^30^ including landslides detection, mapping of urban areas, agricultural lands etc.

RF is a decision tree classifier based on the classification and decision tree (CART)^29^. Each tree is created using a subset of training data through replacement (a bagging approach) and the nodes are split using the best split variable from randomly selected variables. Usually, about two third of the samples (in-bag samples) are used for training the tree, while the remaining one third (out-of-the bag samples) are used for internal cross-validation to estimate the performance of RF algorithm^29^.

The error estimate process of RF model is called as the out-of bag (OOB) error. Each decision tree is independently generated without any pruning and each node is split using a user-defined number of features (Mtry) selected randomly^30^. The algorithm increases the forest by creating trees with high variance and low bias up to a user-defined number of trees (Ntree)^30^. The final decision tree is created by averaging the class assignment probabilities computed for all produced trees. In the process an unsampled data (out-of the-bag samples) is tested against all decision trees generated in the ensemble and each tree is voted for a class membership. The membership class with the majority votes will finally be selected for the tree^29^. It is suggested to use a large number of trees (Ntree) and a relatively small number of split features^30^. For this study, we set the Ntree value of 500 which is acceptable for remote sensing data. The Mtry is set to the default i.e., the square root of the number of input features, which is the standard settings for remote sensing applications.

For different seasons, paddy rice characteristics vary spatially across the Bangladesh and NE India, which induce large variability in the backscatter of Sentinel-1. Therefore, we trained the RF separately for each Boro, Aus and Aman seasons in order to increase the robustness of the algorithms.

### Training and validation data

**Fig. 5:**
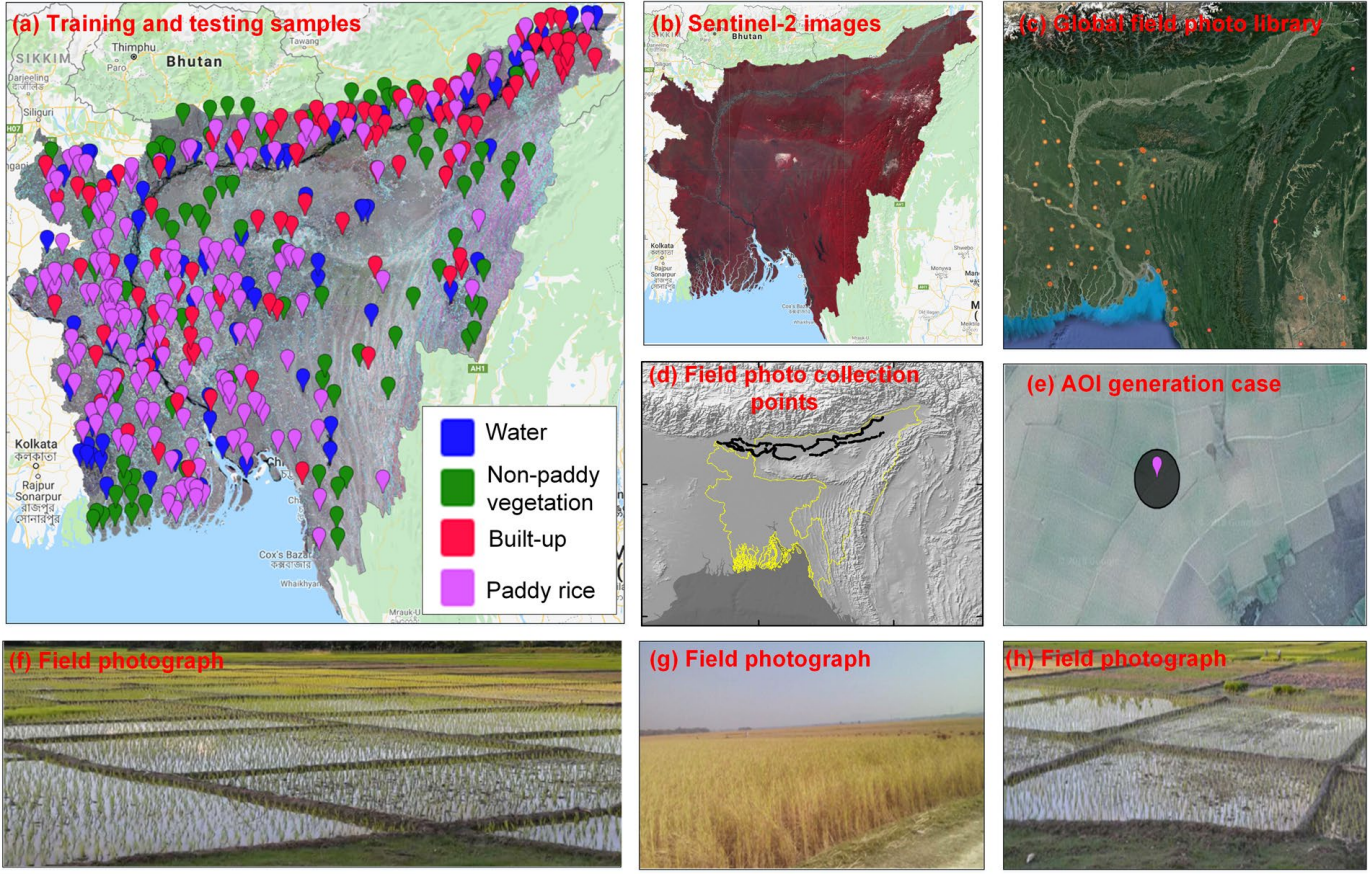
Generation of training and testing samples for RF algorithm. (**a**) Training/testing samples spatial location, the background is Sentinel-1 SAR image; (**b**) Sentinle-2 multispectral image composite during Boro season; (**c**) spatial distribution of field photos from Global Field Photo Library, the cycle sizes represent the photo intensity in the area; (**d**) spatial location of geo-tagged photographs collected during field visit in 2015; (**e**) a circle AOI case of 20 m radius; (**f**–**h**) field photographs.

As our study area is large, to realize a reasonable coverage and correct selection of the training and validation samples (Fig. 5), we collected the reference data from the multiple sources: (1) very high resolution (VHR) images from the Google Earth, (2) Global-Geo-Referenced Field Photo Library (a Google Maps-based scientific field photo management system, (https://www.eomf.ou.edu/photos/), (3) field samples (>2,000) collected during the ground survey in Assam, NE India in May, 2015 by the first author, and (4) visual interpretation of Sentinel-2 multispectral data at 10 m resolution during the mapping season of 2017.

We used the stratified random sampling approach to collect training and validation samples. First, according to the MODIS land cover 2013 products^31^, paddy rice map^12^ of India, and seasonal paddy rice map^11^, the study area was divided into several strata (paddy rice, natural vegetation, water, built-up land, and others). Second, random points were generated in each stratum and then we made areas of interest (AOIs) as circle buffers of the points with the radius of 20 pixels. Third, we checked each AOI and labelled pure land cover AOIs by referring to the mapping year (2017) Sentinel-2 multispectral images (10 m), VHR images of Google Earth, and the field photographs. The AOIs without clear land cover information were omitted. The VHR imagery and field photographs (if available) were used to clarify the land cover types for the AOIs. The field sizes, shapes, and proximities were considered in labelling land cover information of AOIs. Specially, the paddy rice fields can be identified with the characteristics of small field sizes, sharp boundaries and proximity to the river or canals. Finally, a total of 469 AOIs were collected, 133 for vegetation (other crops, forests), 71 for water, 66 for built-up (bare land, dry riverbed), and 199 for paddy rice. The AOIs were distributed uniformly covering the entire study area. The generated AOIs were randomly split into 30/70 percent, 30% was used as training data while the remaining 70% was used for the classification accuracy assessment. No training data were used for the validation.

### Implementation and extraction of paddy rice areas in Google Earth Engine

We mapped paddy rice planting areas accessing the Google Earth Engine platform which hosts multi-petabyte analysis-ready geospatial datasets and provides high-performance computing resources. Mapping seasonal paddy rice using SAR data for a large region requires significant pre-processing steps, high data storage capacity and intense computational power. To extract paddy rice planting areas, the machine learning based RF classifier was fed with the all available time series Sentinel-1 SAR images for the entire Bangladesh and Northeast India for 2017. To make this process efficient the paddy mapping code was written in the JavaScript in the web-based interactive development environment (IDE) (https://code.earthengine.google.com) that utilizes the parallel processing power of Google Earth Engine and the hosted Sentinel-1 SAR data. The classification was performed separately for each of the paddy rice seasons utilizing the generated training AOIs. Finally, the extracted paddy rice maps were exported from the Earth Engine for accuracy assessment and data dissemination.

The SAR-based paddy rice planting area mapping, implemented in GEE includes several benefits. First, reduction of data acquiring and pre-processing time, it would have typically taken a long time to download and pre-process the datasets for a study area like Bangladesh and Northeast India. Second, significant decrease in computational time, the SAR-based RF algorithm inside the GEE, performed the classification of paddy rice fields in a few seconds. With the GEE implementation, such time requirement lessens to negligible, and the method can be quickly extended to larger regions. These high-performance computing resources like GEE facilitate quick and rapid mapping of paddy rice planting areas at a country scale.

## Data Records

The seasonal paddy rice planting area maps are provided for the entire Bangladesh and Northeast India (six states) for 2017. The datasets are available at the figshare repository in a Geotiff format^32^. The dataset is provided in ESPG: 4326 (WGS_1984) spatial reference system. The provided dataset contains map with value 100 and 0 representing paddy rice planting areas and non-paddy rice areas respectively. The dataset extents from 20°44′00′′ to 29°35′40′′N latitude and 88°0′14′′ to 97°39′00′′E longitude. The maps can be viewed and analysed in ArcGIS, QGIS or in similar software.

The dataset of paddy rice planting area will be extended to cover more countries in tropical Asia and involve more detailed information. New versions will be periodically updated and uploaded to the repository upon the availability of new datasets.

## Technical Validation

The validation of our resultant paddy maps includes two folds: (1) validation using the ground truth samples from multiple sources including field survey data and field photographs, visual interpretation with very high resolution images (VHR) and of Sentinel-2 imagery (see section: training and validation data) and (2) comparison with existing rice maps from the International Rice Research Institute (IRRI) and from the analysis of MODIS data^12^. The MODIS-based paddy rice maps in 2017 was derived using the well-established phenology-and pixel-based paddy mapping (PPPM) algorithm that has been successfully used in India and China^33^.

A confusion matrix of paddy rice map was calculated using the collected validation data (see section: training and validation data) to investigate the classification accuracy of the results. The paddy rice maps showed a satisfactory accuracy over 94% (Table 1). The user’s and producer’s accuracies for Boro season rice were 82.4% and 95.0% respectively. The achieved user’s and producer’s accuracies for Aus season rice were 99.4% and 90.6% respectively. The user’s and producer’s accuracies for Aman season rice were 92.8% and 85.97% respectively. The non-rice classes had user’s and producer’s accuracies over 90% in all the three rice seasons. We measured the F-score for paddy rice and non-paddy rice classes to measure the classification performance with respect to precision (user’s accuracy) and recall (producer’s accuracy). The F-score for all the classes exceeds 89%. The commission errors might be caused due to misclassification introduced by small roads, small streams and some non-paddy rice vegetation. The source of omission errors might be introduced from the altered backscatter coefficient values reflected from paddy rice fields due to various environmental factors.

**Table 1:** Confusion matrix of resultant paddy rice planting area maps in three seasons.

Season	Class	Non-Rice	Rice	User’s accuracy	Producer’s accuracy	F-score	Overall accuracy
Boro	Non-rice	906	57	98.4%	94.0%	99.2%	94.2%
	Rice	14	268	82.4%	95.0%	88.2%	
Aus	Non-rice	921	1	98.1%	99. 9%	99.0%	98.3%
	Rice	18	173	99.4%	90.6%	94.8%	
Aman	Non-rice	1032	64	95.9%	98.0%	96.9%	95.2%
	Rice	43	189	92.8%	86.0%	89.3%	

**Fig. 6:**
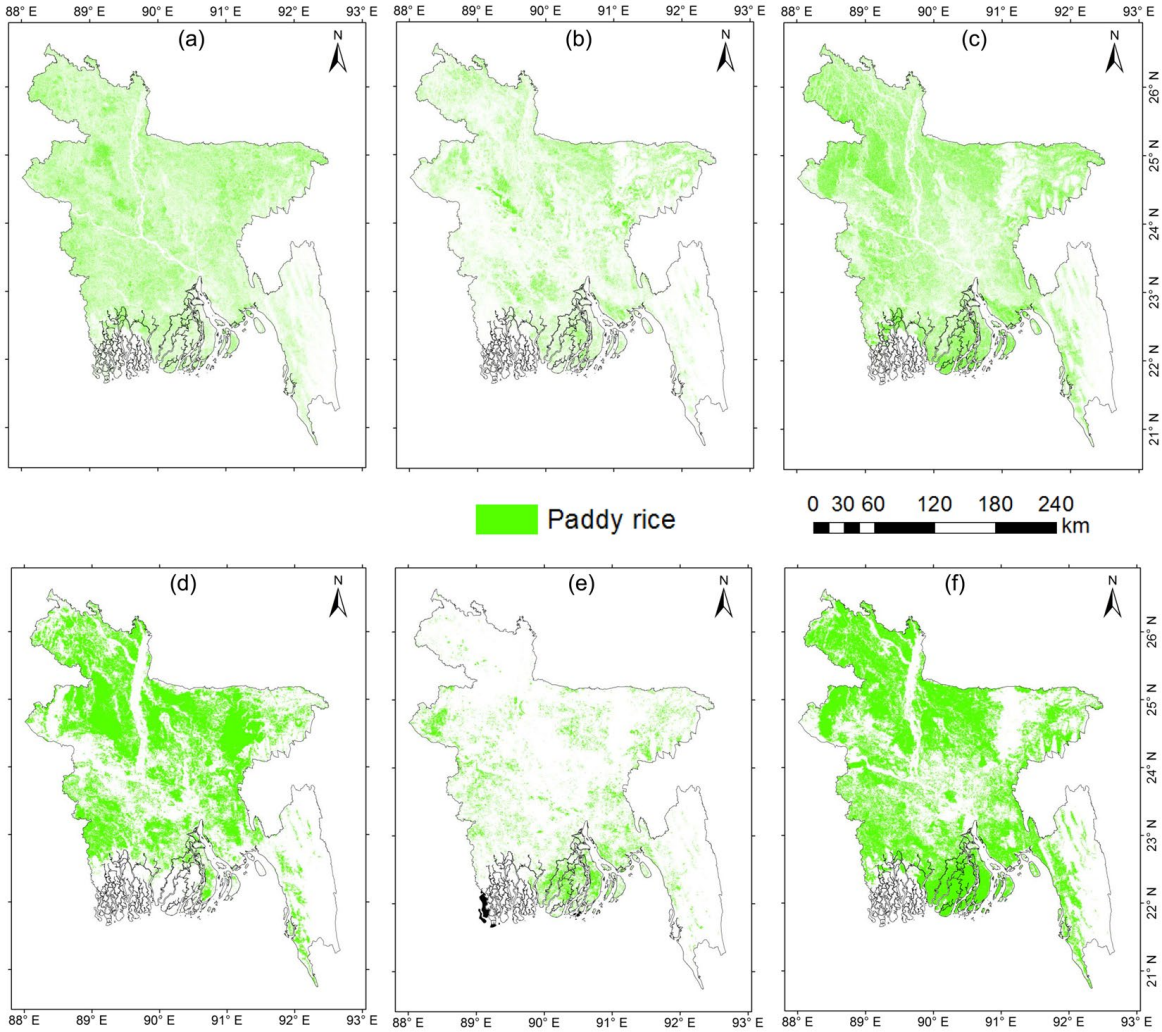
Paddy rice map of Bangladesh in 2017. (**a**–**c**) were derived from analysis of Sentinel-1 images in 2017. (**a**,**d**) Boro season paddy rice; (**b**,**e**) Aus season paddy rice; (**c**,**f**) Aman season paddy rice. (**d**–**f**) are paddy rice map based on IRRI during 2010, showed for comparison purposes. Note that the time periods between the two data are different and the comparison is for a general verification of the rice distribution.

The inter-comparison with the MODIS-based paddy rice maps and the IRRI rice maps showed a consistent spatial pattern between them (Fig. 6). Unlike the coarse resolution MODIS-based paddy rice map, the Sentinel-1 SAR based paddy rice map identified the small paddy rice fields very effectively with its high spatial resolution, evident from the areas near to rivers. Despite the various spatial and temporal resolutions among the products, the comparison can verify the effectiveness of the Sentinel-1 to capture paddy rice pattern and seasonality.

### Uncertainty analysis

There were some potential uncertainty sources that could affect the mapping results. The main uncertainty could be from training samples, small variations in incidence angle, and mixed pixel issues. The landscape of study area is complex and heterogeneous, there may be few wrongly labelled training pixels which may cause misclassification of paddy rice class. The variation of backscatter values due to the incidence angle variation might slightly shift the spatial patterns of paddy rice areas in the region and likely to be a source of mapping uncertainty. The paddy rice fields were relatively small and co-existent with the natural vegetation, seasonal water bodies, and settlements. The mixed-pixels could influence the temporal SAR backscatter profile and cause difficulty in separating paddy rice from the other land cover classes.

## Usage Notes

**Fig. 7:**
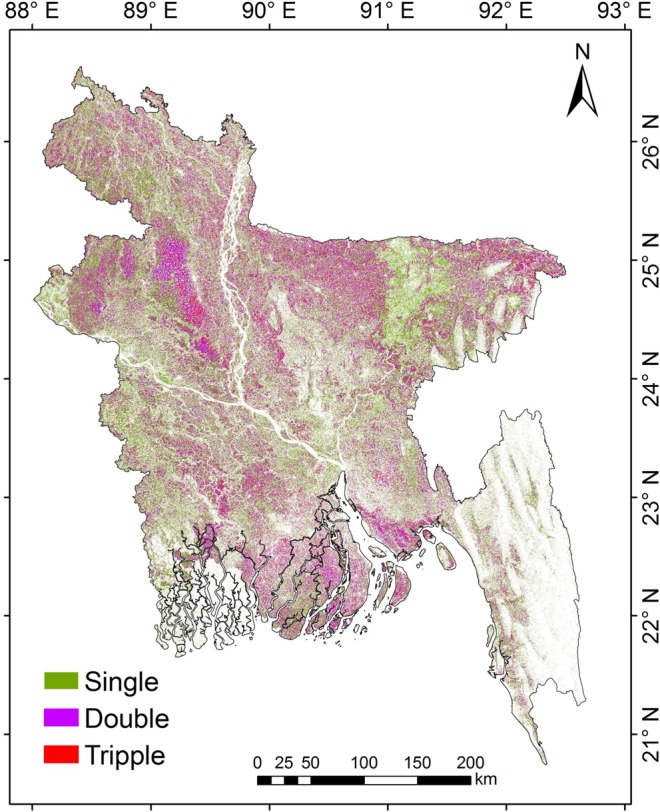
Paddy rice cropping intensity. Paddy rice cropping intensity for Bangladesh during 2017 derived from Sentinel-1 SAR datasets.

A simple but effective approach was presented for mapping paddy rice areas using the VH polarized SAR data, the RF algorithm, and the Google Earth Engine cloud computing environment. The produced maps are provided in high resolution for each rice growing season. The high resolution and seasonal paddy rice maps are essential to study and assessment of regional food security, water uses in paddy rice agriculture, cropping pattern and intensity changes analysis, characterizing spatial and temporal changes in paddy rice planting areas and yields for sustainable agriculture practices, assessment of CH_4_ emissions to the atmosphere caused by paddy rice agriculture. For example, as shown in Fig. 7, the high-resolution paddy rice maps can be used to assess and monitor the intensity of paddy rice planting areas of Bangladesh and NE India. The paddy rice cropping intensity can be identified by simply adding the given seasonal paddy rice maps. It is expected that the intensity map will be of reasonable accuracy as it is generated from the validated rice maps exceeding overall accuracy >90%. However, field-based data were not available with us to verify the cropping intensity pattern. Figure 7 shows that the single and double cropping is mostly common in the country, whereas triple cropping was found in the central Bangladesh during 2017. From the derived paddy rice map, it was found that the Aman and Boro season paddy rice was most widely cultivated in the region, the Aus season rice was cultivated in the regions where the irrigation facilities were available.

## ISA-Tab metadata file

Download metadata file

## Data Availability

JavaScript code used to generate the paddy rice map is available from the figshare repository^32^.
